# The delta-opioid receptor: a therapeutic target for Alzheimer's disease

**DOI:** 10.3389/fnagi.2026.1891339

**Published:** 2026-08-12

**Authors:** Yuan Xu, Wei Guan, Yilin Yang, Yujiao Yang, Ying Xia, Ya Peng

**Affiliations:** 1 Departments of Neurosurgery, The First People's Hospital of Changzhou, Changzhou, Jiangsu, China; 2 Clinical Medical Research Center, The Third Affiliated Hospital of Soochow University, Changzhou, Jiangsu, China; 3 Department of Geriatrics, The First People's Hospital of Changzhou, Changzhou, Jiangsu, China; 4 Shanghai Key Laboratory of Acupuncture Mechanism and Acupoint Function, Fudan University, Shanghai, China; 5 Department of TCM, Shanghai Pudong Hospital, Fudan University Pudong Medical Center, Fudan University, Shanghai, China

**Keywords:** Alzheimer's disease, amyloid-β plaques, microglia, neuroinflammation, tau tangles, δ-opioid receptor

## Abstract

Alzheimer's disease (AD) presents a complex pathogenesis involving amyloid-β (Aβ) plaques, tau tangles, and neuroinflammation. Recent evidence highlights δ-opioid receptor (DOR) as a unique and promising therapeutic target for AD. This review summarizes current knowledge on DOR's multifaceted neuroprotective role in AD pathology. While its involvement in Aβ regulation appears context-dependent, DOR activation consistently attenuates tau hyperphosphorylation, modulates microglial dysfunction, suppresses harmful neuroinflammation and inhibits complement-mediated synaptic pruning. All these findings have been well documented in *in vitro* and *in vivo* AD animal models. Furthermore, preclinical findings from general depression and anxiety models indicate that selective DOR agonists could help alleviate neuropsychiatric symptoms like depression and agitation, which are commonly seen in AD. DOR-associated genetic markers have also shown AD diagnostic potential. We conclude that targeting DOR offers a novel and integrative strategy impacting key AD pathological hallmarks: Aβ, tau, and neuroinflammation, as well as managing cognitive and emotional symptoms. Future research should focus on elucidating precise molecular mechanisms and translating these findings into clinical applications for AD diagnosis and therapy.

## 1 Introduction

Alzheimer's disease (AD) is the leading cause of dementia and is rapidly becoming one of the most burdensome diseases of this century (Scheltens et al., 2021). Although β-amyloid (Aβ) deposition, intracellular neurofibrillary tangles and synaptic loss remain the classical pathological hallmarks of AD (Rostagno, 2022), single-cell transcriptomic studies have revealed that numerous genes associated with AD are enriched in and functionally implicated in microglia, underscoring a critical role of microglia in AD pathogenesis (Scheltens et al., 2021; Merighi et al., 2022; Olah et al., 2020). Substantial efforts have been made in the past several years to expand the ways to diagnose and treat AD. However, recent strategies for both diagnosing and treating AD have revealed several practical problems. From therapeutic perspective, anti-amyloid monoclonal antibodies including lecanemab and donanemab have been reported to alleviate cognitive decline in AD patients, but the actual clinical benefit remains modest, and their application is largely limited to individuals with early-stage, and biomarker confirmed AD (van Dyck et al., 2022; Sims et al., 2023). Traditional AD drugs such as cholinesterase inhibitors and memantine provide only temporary symptomatic benefits without altering disease progression, while several novel approaches, including tau-targeted therapies and anti-inflammatory interventions have failed to demonstrate convincing efficacy in late-phase clinical trials (Cummings et al., 2025). The management of neuropsychiatric symptoms such as depression, agitation, and psychosis, which are common across the course of AD, remains another challenge. They are still treated mainly with off-label psychotropic drugs, which often have limited efficacy and may cause substantial adverse effects in older populations (Tampi and Jeste, 2022). Since AD development is a complex process with the involvements of gene mutations, dysfunctions of different cell types and signal pathways, and the formation of pathological plaques and tau tangles, a systematical modification of these pathological changes can be an optimal solution to slow down or even stop the AD outcomes.

Opioid receptors were originally recognized for their role in modulating pain-associated pathways, but emerging evidence links them to the modulation of pathological and behavioral changes in AD, including cognitive impairment, tau phosphorylation, Aβ production, and neuroinflammation (Torres-Berrio and Nava-Mesa, 2018). Hiller JM et al. (1987); Mathieu-Kia et al. (2001) compared opioid receptor expression in postmortem brains from age-matched controls and patients with AD, and found almost opposite patterns of change of delta-opioid receptor (DOR) and mu-opioid receptor (MOR) in the caudate nucleus and hippocampus. Acetylcholine, as a key neurotransmitters in the central cholinergic system, is essential for learning and memory. By examining the effects of different opioid receptor agonists and antagonists on acetylcholine release, (Feuerstein et al., 1996) reported that acetylcholine release in the human cerebral cortex is mainly regulated by DOR and kappa-opioid receptor (KOR) rather than MOR. Nevertheless, the role of opioid receptors in AD remains insufficiently defined and controversial (Tanguturi and Streicher, 2023; Cai and Ratka, 2012). To clarify that, our group has performed a large amount of *in vitro* and *in vivo* work examining whether opioid receptors regulate AD pathological processes and whether individual receptor subtypes exert distinct effects. These studies identified a unique protective role of DOR in AD (Xu et al., 2025b), a property not observed for other opioid receptor subtypes.

In this review, we focused on the existing clues to show the possible role of DOR in the neuroprotection against AD pathogenesis and addressed the DOR's impact on Aβ plaques, tau tangles, neuroinflammation and microglial dysfunction, as well as psychotropic outcomes. The feasibility analysis of DOR as a potential target for AD pharmacotherapy is conducted in this article. In addition, we explored the potential of DOR as a biomarker for AD diagnosis.

## 2 DOR: discovery, structure, function and pharmacology

DOR, encoded by OPRD1, is one of the three classical subtype of the opioid receptor family, together with MOR and KOR. DOR was first distinguished pharmacologically in 1977, when Lord and his colleagues found that mouse vas deferens exhibited a unique preference for the endogenous opioid peptides Met-enkephalin and Leu-enkephalin (Lord et al., 1977). In late 1992, two labs independently cloned OPRD1 gene from the neuroblastoma-glioma cell line NG-108 (Evans et al., 1992; Kieffer et al., 1992), further confirming DOR as a distinct receptor at the molecular level.

Structurally, DOR belongs to the class A G protein-coupled receptor family. It contains the typical seven-transmembrane helical architecture, with an extracellular N-terminus, three extracellular and intracellular loops, and an intracellular C-terminus. High-resolution structural studies have revealed that DOR shares the conserved opioid receptor binding pocket with MOR and KOR, while subtype-selective ligand recognition depends on differences in residues within the transmembrane domains and extracellular regions (Granier et al., 2012; Fenalti et al., 2014). More recently, (Claff et al., 2019) report the first crystal structures of DOR in an activated state, and further revealed that DOR undergoes significant conformation rearrangements when activated, following by collapse of the sodium pocket and a large rotation of W284^6.58^, which opens space for bulky ligand groups. Upon ligand binding, DOR primarily couples to Gi/o proteins, leading to inhibition of adenylyl cyclase, reduced intracellular cAMP levels, activation of G protein-gated inwardly rectifying potassium channels, and inhibition of voltage-gated calcium channels. These signaling events reduce neuronal excitability and neurotransmitter release (Pradhan et al., 2011). DOR can also recruit β-arrestins, trigger receptor phosphorylation and internalization, and activate downstream pathways such as MAPK/ERK, depending on the ligand and cellular context (Pradhan et al., 2011; Cheng et al., 1998).

Physiologically, DOR is widely expressed in the central nervous system (CNS) and peripheral tissues, including the cortex, striatum, hippocampus, amygdala, olfactory bulb, spinal cord, and dorsal root ganglia with a high density in the cortex and striatum. It has been implicated in pain modulation, mood regulation, emotional responses, learning and memory, seizure susceptibility, and neuroprotection under pathological conditions such as hypoxia or ischemia (Xia, 2015). Furthermore, compared with MOR, DOR activation generally produces less respiratory depression and lower abuse liability, which has made it an attractive target for analgesic and neuropsychiatric drug development (Pradhan et al., 2011; Gendron et al., 2016). A range of endogenous and synthetic ligands have been developed to study DOR function (Table 1). The endogenous ligands for DOR are Met-enkephalin, Leu-enkephalin. Selective synthetic experimental agonists include [D-Pen^2^, D-Pen^5^]enkephalin (DPDPE), [D-Ala^2^, D-Leu^5^]-Enkephalin (DADLE), SNC80, BW373U86, deltorphin II (DLT-II), and the clinical candidates ADL5859 and ADL574, although these were discontinued partly because of pro-convulsant activity (Fujii et al., 2013; Nagase and Saitoh, 2019). Our group has long used a unique DOR agonist, UFP-512, which has a high selective and potent binding affinity for DOR (Polo et al., 2019). More recently, biased DOR agonists that preferentially activate G protein pathways over β-arrestin recruitment-PN6047 have been developed to dissociate analgesia from convulsions (Asghar et al., 2022). Moreover, DOR antagonists include naltrindole, naltriben, 7-benzylidenenaltrexone, BNTX are widely used in *in vitro* and *in vivo* DOR studies (Fujii et al., 2013). Radioligands like [^3^H]naltrindole and the PET tracer [^11^C]methylnaltrindole are also broadly used for receptor autoradiography and *in vivo* imaging (Yamamura et al., 1992; Madar et al., 2007). These pharmacological tools have greatly advanced the understanding of DOR biology, although the receptor's disease-specific functions, including its potential involvement in AD-related pathology and neuropsychiatric symptoms, remain incompletely defined.

**Table 1:** Commonly used DOR ligands.

Compound	Type	Structural features	Common experimental applications
DOR agonists			
Met-enkephalin	Endogenous peptide agonist	Pentapeptide (Tyr-Gly-Gly-Phe-Met)	DOR/MOR signaling, neuroprotection, immunomodulation, pain research. (i.c.v/i.t./local brain-region microinjection/*in vitro* administration)
Leu-enkephalin	Endogenous peptide agonist	Pentapeptide (Tyr Gly Gly Phe Leu)	DOR/MOR signaling, nociception, and dynorphin system-related research. (i.c.v/i.t./local brain-region microinjection/*in vitro* administration)
DPDPE	Synthetic peptide agonist	Cyclic pentapeptide containing a D-Pen^2^, D-Pen^5^ disulfide bridge	analgesia, neuroprotection, ischemic tolerance, emotional behavior, cognitive function, and neuroinflammation research (i.c.v/i.t./local brain-region microinjection/*in vitro* administration)
DADLE	Synthetic peptide agonist	pentapeptide analog of enkephalin, [D-Ala^2^, D-Leu^5^]-enkephalin	DOR signaling, neuroprotection, ischemia/hypoxia tolerance, cardioprotection, pain research, neuroinflammation (i.c.v./i.t./local brain-region microinjection/systemic injection/*in vitro* administration
KK-103	Synthetic peptide agonist	Derived from Met-enkephalin-related sequences	DOR/MOR-related opioid signaling, analgesia and pain research, neuroprotection, peptide opioid pharmacology (i.c.v./i.t./local brain-region microinjection/*in vitro* administration)
Deltorphin I/II	Natural peptide agonist	Heptapeptide isolated from frog skin, containing D-amino acids	DOR analgesia, neuroprotection, ischemia/hypoxia, and behavioral research. (i.c.v./i.t./local brain-region microinjection/*in vitro* administration)
BW373U86	Synthetic non-peptide agonist	Diarylmethylpiperazine derivative; brain-penetrant	DOR signaling, antinociception, antidepressant-like effects, convulsion/seizure liability, receptor internalization, and behavioral pharmacology. (systemic administration/i.c.v./local brain-region microinjection/*in vitro* administration)
SNC80	Synthetic non-peptide agonist	Diarylmethylpiperazine derivative; highly selective, brain-penetrant	DOR-mediated antinociception, antidepressant-like and anxiolytic-like behavior, seizure/convulsion liability, receptor trafficking/internalization, reward and locomotor studies (systemic administration/i.c.v./local microinjection/*in vitro* treatment)
UFP-512	Synthetic non-peptide agonist	Diarylmethylpiperazine derivative; highly selective, brain-penetrant	DOR signaling, antinociception, antidepressant-like effects, neuroprotection, receptor desensitization/internalization, and behavioral studies (systemic administration/i.c.v./local injection/*in vitro* application)
ADL5859	Synthetic non-peptide agonist	Orally bioavailable, selective DOR agonist; small-molecule analgesic candidate	Chronic pain, inflammatory pain, neuropathic pain, osteoarthritis pain, antinociception, and behavioral safety profiling (oral/systemic/*in vitro* administration)
ADL5747	Synthetic non-peptide agonist	Orally bioavailable, selective DOR agonist; small-molecule analgesic candidate	Inflammatory pain, neuropathic pain, chronic pain, visceral pain, and antinociceptive efficacy with reduced classical opioid-like side effects (oral/systemic/*in vitro* administration)
PN6047	Synthetic non-peptide agonist; biased agonist	Biased toward G protein signaling; structure not fully disclosed	DOR signaling bias, antinociception, chronic pain models, receptor trafficking, β-arrestin/G protein pathway comparison, and side-effect profiling (systemic administration/*in vitro* application)
TAN-67	Synthetic non-peptide agonist	Benzofuran-based non-peptide ligand; conformationally constrained structure	Pharmacological activation of DOR signaling; studies of DOR-mediated neuroprotection, analgesia, seizure modulation, emotional behavior, and cardiovascular/ischemic injury responses (systemic injection/i.c.v./i.t. injection/local brain-region microinjection/*in vitro* application)
KNT-127	Synthetic non-peptide agonist	small-molecule benzazepine derivative; highly selective for DOR	Antidepressant- and anxiolytic-behavioral studies, pain/analgesia research, seizure and neuroprotection models (systemic administration/i.c.v. injection/local brain-region microinjection/*in vitro* administration
DOR antagonists			
Naltrindole	Synthetic antagonist	Naltrexone-derived indole compound; highly selective	Pharmacological blockade of DOR, validation of DOR-mediated effects, pain, mood, addiction, neuroprotection, and receptor subtype studies (systemic administration/i.c.v./i.t./local injection/*in vitro* application)
Naltriben	Synthetic antagonist; δ2-preferring antagonist	Benzofuran analog of naltrindole; preferential antagonist activity at putative δ2-opioid receptor subtype	Differentiation of δ1/δ2 DOR pharmacology, pain and antinociception studies, seizure liability, behavioral pharmacology, and DOR mechanism validation (systemic administration/i.c.v./i.t./local injection/*in vitro* application)
BNTX	Synthetic antagonist; δ1-preferring antagonist	7-benzylidenenaltrexone derivative; preferential antagonist activity at putative δ1-opioid receptor subtype	Differentiation of δ1/δ2 DOR pharmacology, antinociception, behavioral assays, receptor subtype mapping, and validation of DOR-mediated mechanisms (systemic administration/i.c.v./i.t./local injection/*in vitro* application)
BMS-986187	Synthetic positive allosteric modulator of DOR	7-Benzylidenenaltrexone derivative, δ1-selective	DOR allosteric modulation, probe of endogenous enkephalin signaling, pain research, receptor pharmacology, signaling bias, and *in vitro* DOR assay development (commonly used *in vitro* and in some systemic/*in vivo* pharmacology studies)
[^3^H]naltrindole	Radiolabeled DOR antagonist/radioligand	Tritium-labeled naltrindole; high-affinity, selective radioligand	DOR binding assays, receptor density quantification, saturation and competition binding, autoradiography, ligand affinity profiling, and validation of DOR expression in brain tissue, spinal cord, or transfected cells (*in vitro* administration)
[^11^C]methylnaltrindole	PET radiotracer/radiolabeled DOR antagonist	Carbon-11-labeled methylated naltrindole derivative; designed for *in vivo* PET imaging	PET imaging of δ-opioid receptor distribution and occupancy, *in vivo* receptor mapping, CNS pharmacodynamic studies, occupancy studies with DOR ligands, and translational neuroimaging (intravenous injection for small-animal or human PET imaging)

## 3 DOR's involvement in Aβ regulation

Previous research presented conflicting evidence regarding the role of DOR in Aβ regulation (Sarajärvi et al., 2011, 2015; Min et al., 2018; Teng et al., 2010; Xu et al., 2020; Ni et al., 2006). While some studies indicated that DOR activation promotes Aβ generation, others suggested that its dysfunction acts as a risk factor for Aβ deposition. Ni et al. demonstrated that activating DOR using DOR agonist DADLE enhances Aβ production and secretion in C99-transfected cells and primary hippocampal cultures, likely by facilitating gamma-secretase-mediated cleavage of the Aβ precursor C99 (Ni et al., 2006). Teng et al. (2010) further proposed that DOR activation forms a complex with BACE1 and gamma-secretase, promoting Aβ precursor protein (APP) processing and Aβ generation through trafficking into late endosomes/lysosomes. Conversely, DOR knockdown or inhibition in APP/PS1 mice reduces Aβ production and associated cognitive deficits. However, it is important to note that DADLE, as well as other early enkephalin-derived agonists, possesses a significant affinity for the MOR in addition to DOR (Raynor et al., 1994; Cassell et al., 2019). Therefore, the conclusions drawn from these pharmacological experiments may be confounded by simultaneous MOR activation or other off-target effects, leading to uncertainty about the specific role of DOR in Aβ regulation.

Consistent with this concern, a genetic study about DOR has yielded a totally different outcome. A genetic variation at DOR's Cys27 residue, which impairs receptor maturation and surface trafficking, is considered a loss-of-function mechanism (Leskelä et al., 2007). Paradoxically, this loss-of-function variant significantly increases APP processing and Aβ generation in SH-SY5Y and HEK293 cells (Leskelä et al., 2009, 2012), a result that directly contrasts with the Aβ-promoting effect of DOR activation reported by Ni et al. and Teng et al. This discrepancy further highlights the need to consider the selectivity of DOR ligands when designing experiments, as well as the demand to develop more selective ligands to clarify DOR's function.

Recent studies employing more specific DOR agonists have begun to resolve these discrepancies (Min et al., 2018; Xu et al., 2020). In an ischemia/reperfusion (I/R) mouse model, post-ischemic administration of the selective non-peptide agonist Tan-67 improved survival and neurobehavioral outcomes. Although initially increasing APP expression, maturation and processing within the first 6 h post-I/R, Tan-67 conversely attenuated BACE1 expression and activity by 24 h following I/R, leading to reduced Aβ production, and the effect was totally abolished by DOR antagonist naltrindole (Min et al., 2018). More recently, our research group applied the specific and potent DOR agonist UFP-512 to Aβ oligomer-treated PC12 cells and APPswe SH-SY5Y cells, we have also demonstrated that DOR is protective against AD pathology by inhibiting BACE1 expression/activity, attenuating APP cleavage, and reducing Aβ generation (Xu et al., 2020). Such protection could be reversed by DOR antagonism, and DOR knockdown increased BACE1 activity and accelerated APP processing (Xu et al., 2019). *In vivo*, 9-month-old APP/PS1 mice treated intraperitoneally with UFP-512 for 1 week showed a sharp decrease in insoluble Aβ42 levels in cortex and hippocampus, and a 39.7% reduction in Aβ plaque burden compared to saline-treated controls (Xu et al., 2025b).

**Figure 1:**
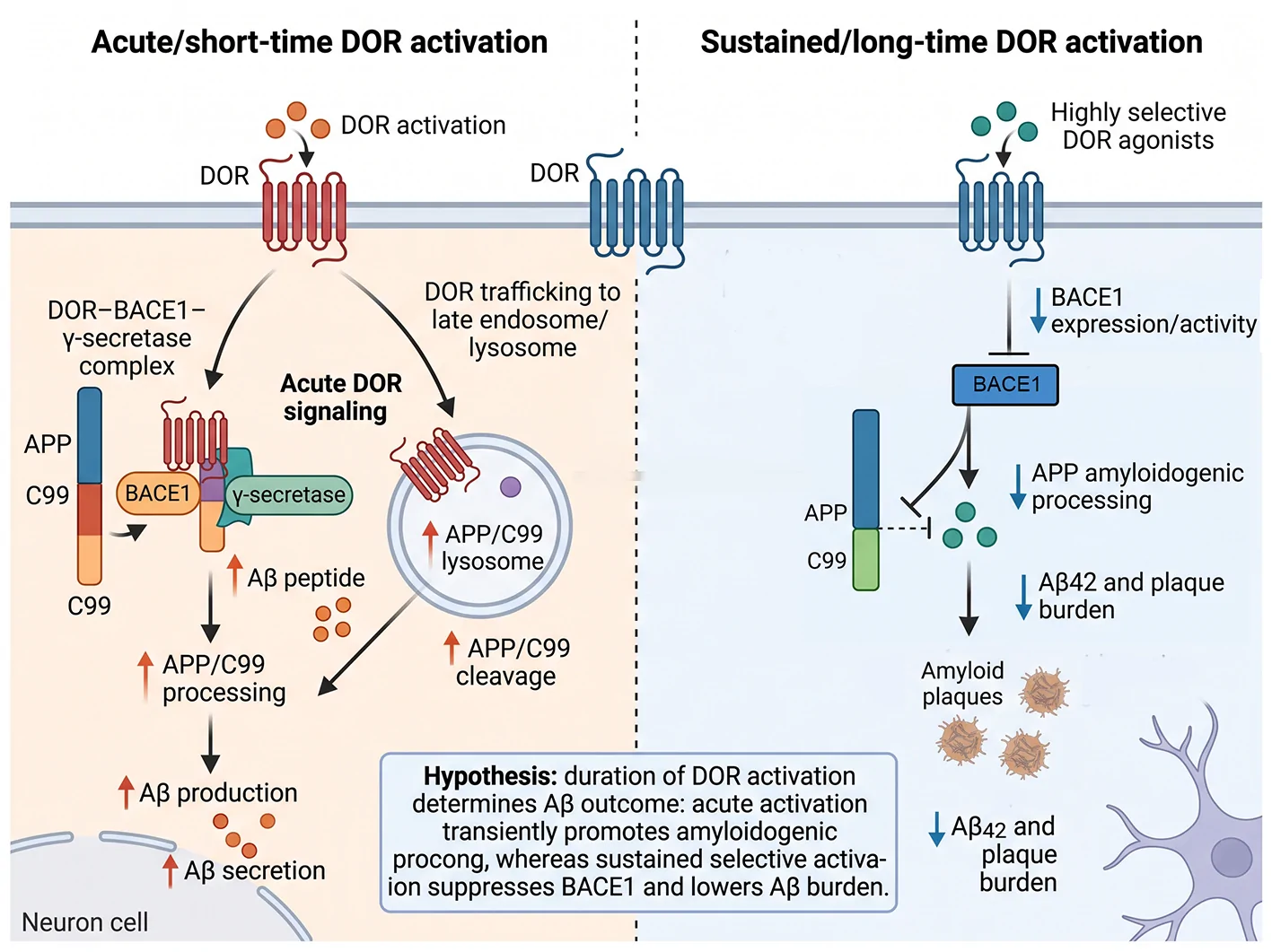
Schematic illustration of the time-dependent effects of DOR activation on Aβ pathology. **Left panel** acute/short-time DOR activation promotes amyloidogenic processing. Binding of DOR agonists triggers DOR–BACE1-γ-secretase complex formation and DOR trafficking to late endosomes/lysosomes. This signaling cascade enhances APP/C99 cleavage, leading to a marked increase in Aβ peptide production and secretion. **Right panel** sustained/long-time DOR activation exerts neuroprotective effects. Highly selective DOR agonists suppress BACE1 expression and activity, which in turn attenuates APP amyloidogenic processing. This results in decreased Aβ42 generation, reduced insoluble Aβ42 accumulation, and lower amyloid plaque burden.

These contrasting findings can be explained by considering experimental variables, including agonist selectivity, model systems, treatment duration, and experimental conditions, which underscore the complex, context-dependent role of DOR in Aβ regulation. Early studies mainly relied on agonists with limited DOR selectivity, whereas subsequent work employed highly specific ligands (Tan-67, UFP-512, etc.) that minimize off-target interference. The model systems also differ, ranging from transfected cell lines to transgenic mice and I/R models, which likely engage distinct downstream signaling cascades. Crucially, emerging evidence reveals a profound time-dependent effects of DOR activation on Aβ regulation: short-term exposure (minutes to 6 h) appears to increase APP processing and Aβ generation, whereas sustained activation (24–48 h) consistently inhibits BACE1 activity, reduces Aβ production, and attenuates plaque formation *in vivo* (Min et al., 2018; Xu et al., 2020). We therefore propose the hypothesis that the duration of DOR activation is a critical determinant of its effect on Aβ regulation, with acute activation transiently promoting amyloidogenic processing and prolonged activation driving neuroprotective, Aβ-lowering pathways (Figure 1). However, this time-dependent model currently remains a hypothesis that requires validation through studies specifically designed to compare different treatment durations under identical conditions. Future therapeutic evaluation of DOR-targeted therapies requires careful consideration of agonist selectivity, dosing regimens and longitudinal assessment windows to harness its beneficial, long-term inhibitory effects on Aβ accumulation and AD progression.

## 4 The regulation of DOR in tau disorders

Few studies have investigated the role of DOR in tau-related disorders. Our group initiated this work in 2023, first demonstrating that DOR activation leads to significant neuroprotection against tau hyperphosphorylation, a core pathological feature of AD (Li et al., 2025). In an okadaic acid (OA)-induced tauopathy model using highly differentiated PC12 cells, DOR activation with the specific agonist UFP-512 dose-dependently reduced tau hyperphosphorylation at Thr231 and Ser262 sites, which could be reversed by the DOR antagonist naltrindole. Mechanistically, DOR activation attenuated tau pathology by downregulating cyclin-dependent kinase 5 (CDK5) and suppressed AMP-activated protein kinase (AMPK) phosphorylation at Thr172, pathways directly implicated in aberrant tau phosphorylation. Furthermore, DOR activation mitigated downstream neuronal injury by reducing the expression of cell cycle reactivation markers (CyclinD1 and CyclinB1) and apoptosis indicators (cleaved caspase-3) (Li et al., 2025), suggesting that it disrupts the link between tau hyperphosphorylation, aberrant cell cycle re-entry, and neuronal death. However, it must be noted that these findings are based solely on a rodent pheochromocytoma-derived PC12 cell line, representing a major limitation. Further validation in human neuronal models, such as SH-SY5Y cells or iPSC-derived neurons, and *in vivo* tauopathy/AD models, is therefore explicitly needed to confirm the translational relevance of these mechanisms. Additionally, exploring the precise regulatory interplay between DOR signaling and CDK5/AMPK is critical, including investigating DOR's impact on CDK5 activators (p35/p25 cleavage) and AMPK upstream regulators.

**Figure 2:**
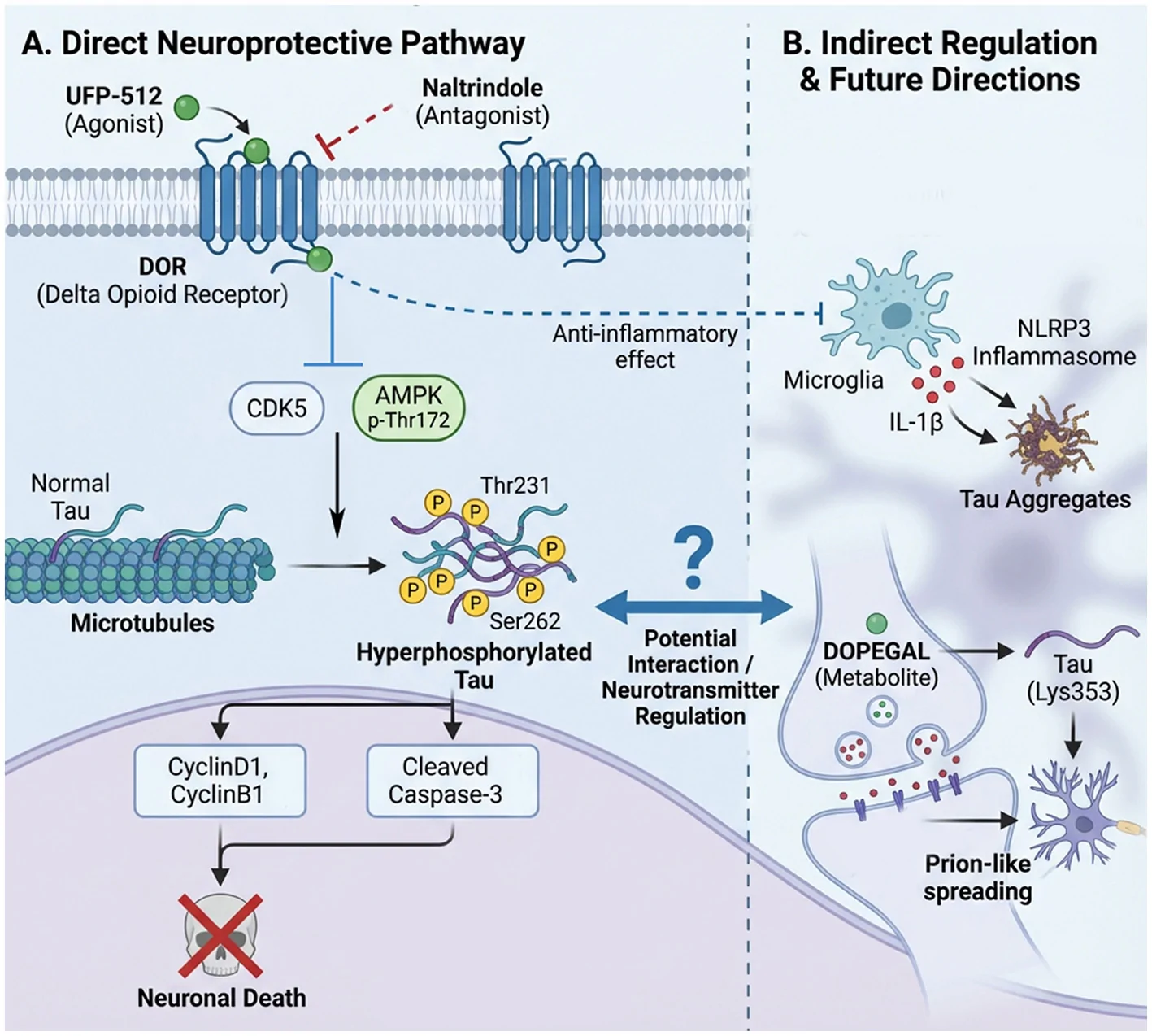
Proposed mechanisms of DOR-mediated neuroprotection against tau pathology. **(A)** Direct pathway. In highly differentiated PC12 cells mimicked AD model, DOR activation by the agonist UFP-512 suppresses tau-related pathological signaling, whereas the DOR antagonist naltrindole blocks DOR activation and reverses the protective effects of UFP-512. DOR activation reduces CDK5 and AMPK activity, as indicated by decreased AMPK phosphorylation at Thr172, thereby attenuating tau hyperphosphorylation at Thr231 and Ser262. This reduction in tau hyperphosphorylation is associated with decreased levels of aberrant cell-cycle re-entry markers (cyclin D1 and cyclin B1) and cleaved caspase-3, a marker of apoptosis. Consequently, DOR activation inhibits tau pathology-induced neuronal death. **(B)** Indirect and potential pathways. DOR may also mitigate tau pathology via its anti-inflammatory properties by suppressing microglial NLRP3 activation and IL-1β release. Additionally, DOR signaling might intersect with the DOPEGAL-mediated “prion-like” spread of tau through neurotransmitter regulation. Symbols: black solid arrows indicate activation or promotion; blue T-shaped bars indicate DOR-mediated inhibition or suppression; red T-shaped bars indicate pharmacological blockade by naltrindole; blue dashed arrows indicate proposed or hypothetical DOR-mediated inhibition.

Beyond directly suppressing tau hyperphosphorylation, DOR's anti-inflammatory properties (Xu et al., 2023, 2025b; Yoshioka et al., 2023) may indirectly ameliorate tau pathology, as chronic neuroinflammation such as microglial NLRP3 inflammasome activation and IL-1β release exacerbates tau aggregation and propagation (Chen and Yu, 2023; Davidson et al., 2023). Notably, it was reported that DOPEGAL, a norepinephrine metabolite in the locus coeruleus, covalently modifies tau at Lys353 residue, accelerating its “prion-like” spread (Kang et al., 2022). DOR signaling may intersect with this pathway, considering its role in regulating neurotransmitter systems and stress responses (Xia, 2015; Tkaczynski et al., 2022; Nakamoto and Tokuyama, 2023). These mechanistic insights highlight critical future directions: elucidating DOR's impact on tau seeding and transmission, characterizing its interaction with tau strains in human-derived models, and developing combinatorial strategies targeting tau oligomer-induced cellular senescence and neuroinflammation (Figure 2).

## 5 DOR-mediated microglial modulations

**Figure 3:**
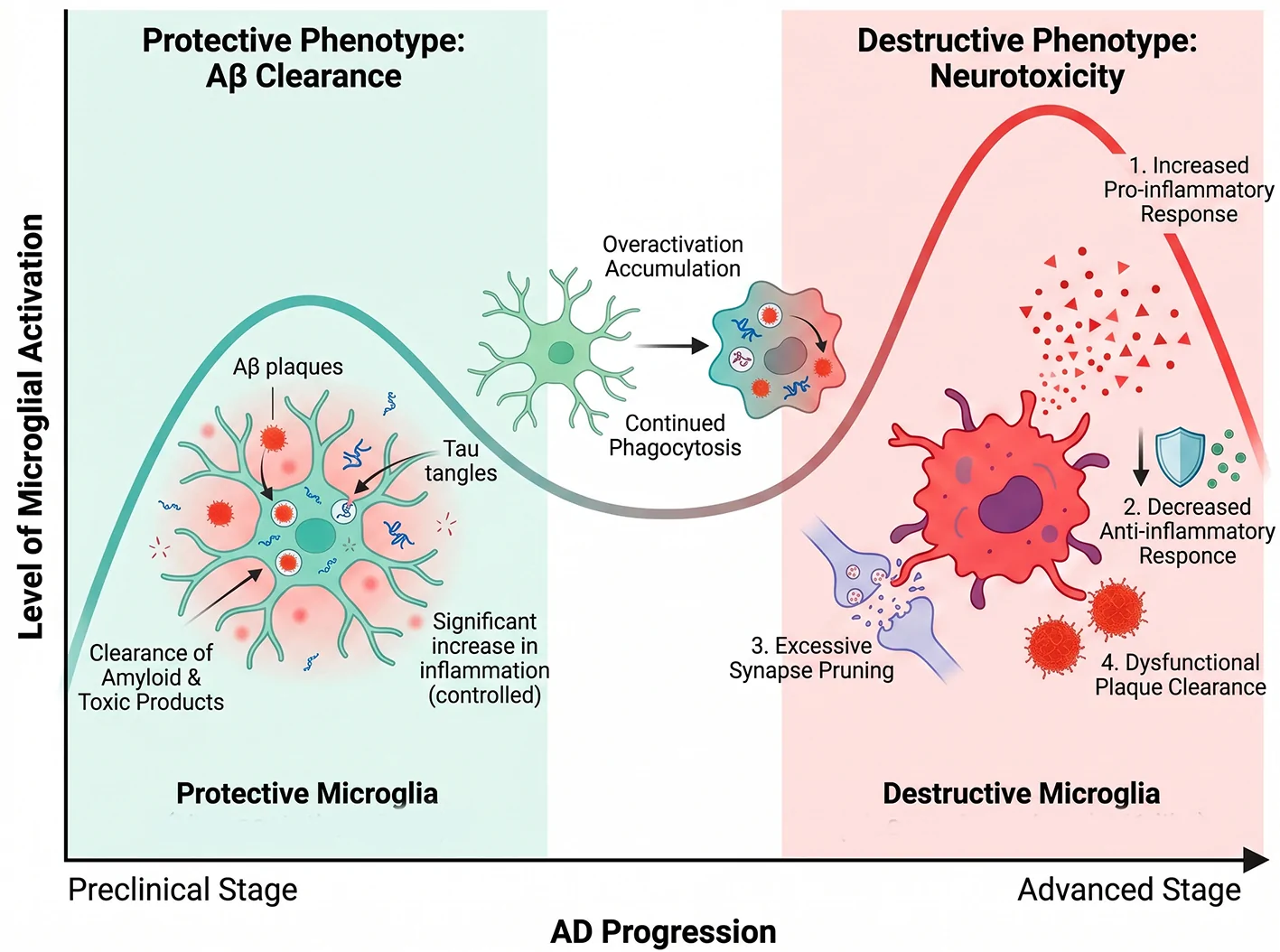
Biphasic activation and functional transformation of microglia in AD. Schematic representation of the biphasic microglia activation in AD. The first peak features protective microglia clearing Aβ, appearing in the preclinical stage of AD. Chronic burden leads to the second, higher peak in the advanced stage of AD, which is characterized by increased pro-inflammatory responses, excessive synapse pruning and dysfunctional plaque clearance.

Recent studies have implicated the critical role of microglia in neuroinflammation, synaptic plasticity, learning and memory, which are involved in AD pathogenesis (Zhang et al., 2024; Früholz and Meyer-Luehmann, 2024). More than one-third of the high-risk genes for AD are expressed by microglia and are associated with microglial function and innate immune responses (Chen and Holtzman, 2024; Fan et al., 2024), further highlighting the close linkage between microglia and AD risk. When microglia activated, they not only serve as inflammatory mediators to release proinflammatory factors and kill pathogens, but also function as phagocytes to remove debris, injured synapses or aggregated proteins (Merighi et al., 2022; Zhang et al., 2024; Wright-Jin and Gutmann, 2019; Borst et al., 2021). However, aberrant and sustained activation of microglia can trigger and worsen AD. It has been reported that there are two peaks of microglial activation during AD development (Fan et al., 2017). The first peak occurs in preclinical stages of AD, appearing as a significant increase in inflammation and the presence of Aβ deposits in activated microglia, which was suggested as a protective phenotype trying to clear amyloid and other toxic products (Fan et al., 2017). The continued phagocytosis of tau and Aβ by microglia contributes to the overactivation of microglia, leading to the second peak of microglia activation during the advanced stage of AD (Fan et al., 2024). At this stage, microglia triggers increased pro-inflammatory responses, decreased anti-inflammatory responses, excessive synapses pruning and dysfunctional AD plaques clearance (Merighi et al., 2022; Fan et al., 2017, 2024). The role of microglia is transformed from a protective one to a destructive one (Figure 3). Therefore, inhibiting microglial overactivation are new target directions for AD therapy, especially at the late stage of AD.

### 5.1 DOR protection against microglia-mediated neuroinflammation

Substantial research indicates the role of DOR in the control of microglial activation and microglial-mediated neuroinflammation (Xu et al., 2022, 2023; Sawada and Yamakage, 2024; Vicario et al., 2022; Cheng et al., 2021). In both human and mouse microglia, DOR activation was found to significantly inhibit pro-inflammatory cytokines release and superoxide production (Xu et al., 2022, 2023; Vicario et al., 2022; Cheng et al., 2021). A work conducted in pregnant C57BL/6JJmsSlc female mice showed that pregnancy-induced analgesia suppressed activation of microglia, associating with upregulating of DOR in the anterior cingulate cortex in late-pregnant mice (Sawada and Yamakage, 2024). Indeed, our recent research has investigated the relationship between DOR and microglia in detail. Our work showed DOR could be largely upregulated by IL4 and hypoxia in BV2 microglia cell line, but downregulated by LPS and Aβ1-42 oligomer (Xu et al., 2022, 2025a). DOR activation with UFP-512 suppressed BV2 activation and its inflammatory responses under LPS injury, hypoxic injury and AD injury, while DOR antagonism, enhanced microglial activation in all of these conditions (Xu et al., 2022, 2025a). Genetic studies and *in vivo* work further validated these conclusions. In APP/PS1 mouse model, the administration of DOR agonist UFP-512 largely reduced the neuroinflammation in AD mouse brain, as well as inhibited microglial activation and macrophage infiltrations (Xu et al., 2025b). Genetically overexpressing DOR in BV2 cells upregulated enriched microglial homeostasis genes and downregulated disease associated microglial (DAM) genes with an inhibition of microglial activation, while knocking down DOR induced an opposite effect (Xu et al., 2022, 2025b). Taken together, all these studies strongly demonstrated a negative regulation of DOR in microglial activation and its related neuroinflammation.

To further explore the underlying mechanism regarding DOR-mediated microglial regulations, the research was performed at molecular, protein and cellular levels. Mounting evidence suggests that caspase 3 act as the mediator of let-7c-5p in regulating microglial activation and neuroinflammation, which is independently from its apoptotic inducer character (Ni et al., 2015; Lv et al., 2018). DOR activation was well demonstrated to reduce caspase 3 expression under several situations (Yang et al., 2009; Xu et al., 2016), which may potentially regulate the microglia through caspase signaling (Chen et al., 2020). This conjecture was validated by Cheng's work. They showed that DOR activation suppressed microglia pro-inflammatory activation and inhibited LPS-induced inflammation via the mitogen-activated protein kinase (MAPK)/caspase-3 pathway in BV2 cell line (Cheng et al., 2021). In AD mimicked microglia, RNA-seq revealed that the OPRD1 gene encoding DOR is negatively correlated with HMGB1 mRNA contents (Xu et al., 2025b). Indeed, we observed that DOR activation down-regulated HMGB1/NF-κB signaling pathway both in AD mimicked BV2 cells and AD mouse brains with a major effect targeting microglia. Moreover, in a microglia-neuron coculture system, overexpressing DOR in microglia also inhibited the neuroinflammation induced by AD and saved neurons through microglia-neuron crosstalk, with a significant decrease in extracellular HMGB1 release (Xu et al., 2025b), further suggesting DOR-mediated neuroprotection is partly dependent on DOR's modulation of microglia by targeting HMGB1-NF-κB signaling pathway.

### 5.2 DOR protects from microglia-mediated synapses pruning

More recently, our group also identified DOR as a novel classical complement pathway (CCP) inhibitor via interacting with C1q (Xu et al., 2025a). C1qa, C1qb, C1qc constitute the C1q, which is mainly produced by microglia (Lui et al., 2016; Hong et al., 2016). There is a tight negative correlation between DOR and C1q in microglia both at the molecular level and the protein level. Besides that, DOR can bind to C1q to form complexes especially when it was activated (Xu et al., 2025a). Indeed, we have found that DOR activation or overexpression largely restrained CCP activation, attenuated microglial phagocytosis, and rescued synaptic proteins in AD pathologies (Xu et al., 2025a). Since upregulated C1q was observed in the cerebrospinal fluid (CSF) of patients with dementia (Chatterjee et al., 2023), and genetically deletion or inhibition of C1q was reported to effectively reduce microglia-mediated aberrant synaptic loss and thus restored cognitive function in aged AD mice (Hong et al., 2016; Dejanovic et al., 2022; Lv et al., 2024), our findings support a beneficial role of DOR against AD pathology by modulating microglia-mediated synapses pruning mechanism through the crosstalk with C1q.

**Figure 4:**
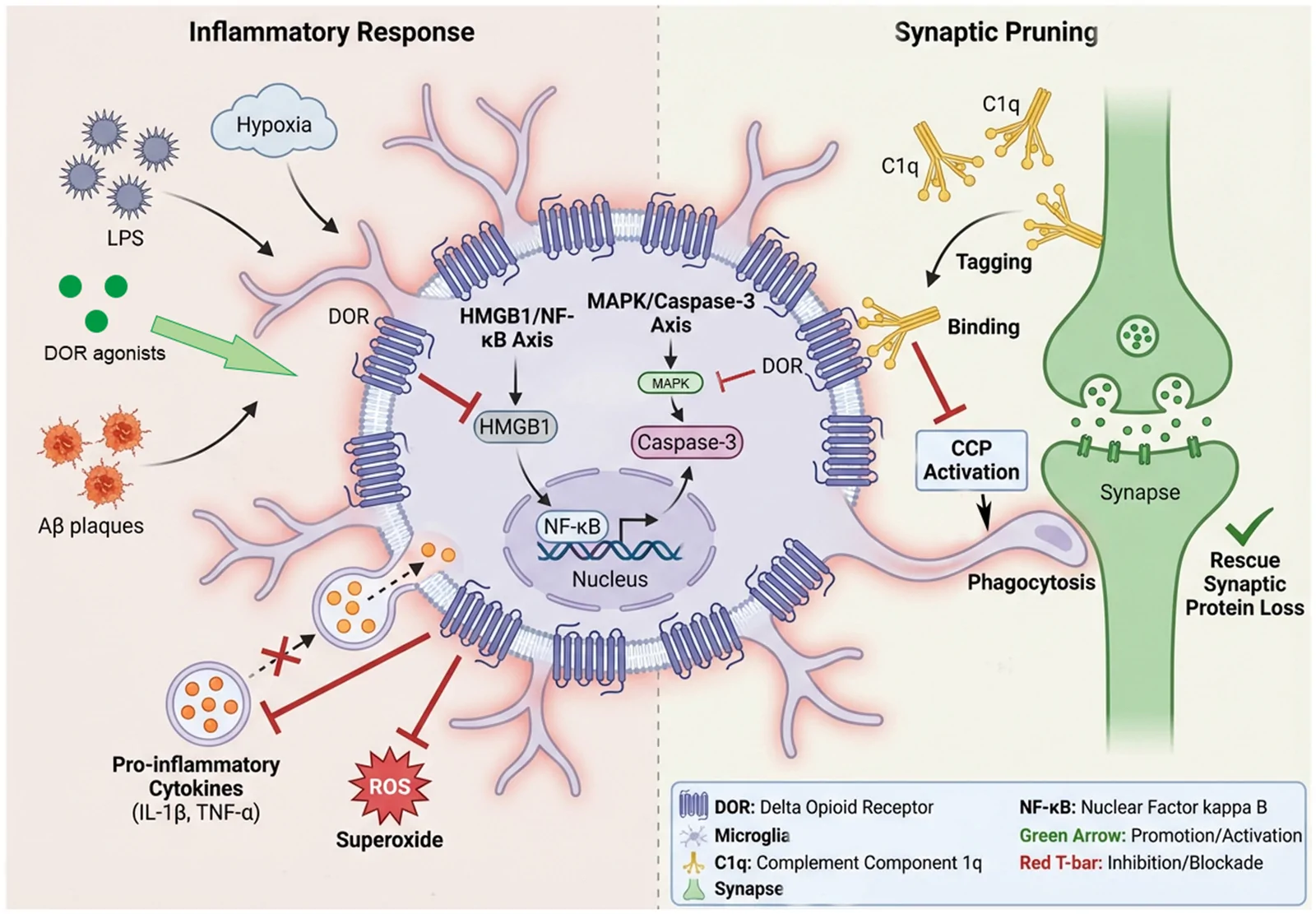
DOR-mediated modulation of microglial responses: a dual mechanism against neuroinflammation and synaptic loss. **Left panel**: suppression of neuroinflammation. Under pathological stimuli such as Aβ oligomer, LPS and hypoxia, DOR activation negatively regulates the MAPK/caspase-3 and HMGB1/NF-κB signaling pathways. This negative regulation significantly reduces the secretion of pro-inflammatory cytokines and superoxide production. **Right panel**: inhibition of synaptic pruning. DOR acts as a novel inhibitor of the CCP. By directly binding to microglial-derived C1q, DOR prevents aberrant CCP activation and subsequent microglial phagocytosis of synapses, thereby rescuing synaptic from loss.

Activated microglia is likely to be a double-edged sword in AD pathogenesis. On one hand, activated microglia constantly monitors the brain microenvironments and clear extracellular Aβ. On the other hand, microglia triggers neuroinflammation and synaptic loss, which aggravates AD development (Lv et al., 2024). It is worth noting that DOR, as a potential target to modulate microglia, it selectively prevents synapse loss and inhibited neuroinflammation induced by microglia activation (Xu et al., 2022, 2025a, b; Cheng et al., 2021) (Figure 4), but does not promote Aβ plaque formation (Min et al., 2018; Xu et al., 2020). These properties make DOR a potential therapeutic target for AD. The major mechanism underlying its action on AD is, at least partly, dependent on microglial regulation, though it needs further investigation.

## 6 DOR-associated biomarkers for AD diagnosis

In addition to AD therapy, early and accurate diagnosis of AD is important for the patients and caregivers, which enable both the patient and family to plan for the future and make appropriate changes to maintain life quality for longer time (Porsteinsson et al., 2021). Two fundamental questions to be solved in the developments of biomarker diagnosis are how to label AD more specifically excludes from other dementia, and how to label AD before clinical symptomatology. Aberrant DNA methylation has been observed both in brain samples and peripheral blood samples with AD as a promising marker to monitor the progression of disease (Nikolac Perkovic et al., 2021; De Plano et al., 2024; Wei et al., 2020). Ji et al. (2017) compared the DNA methylation levels of OPRD1 (gene encodes DOR) promoter CpG sites from the blood samples of 51 AD cases and 63 controls, and found that CpG3 methylation of OPRD1 significantly upregulated in AD cases. A neuroimaging and genetic analysis of 738 elderly people also revealed that the OPRD1 variant was associated with reduced brain volumes, and neurodegenerative prediction (Roussotte et al., 2014). Although these two studies shed light for the OPRD1 potential as an AD diagnostic biomarker, whether OPRD1 can be a qualified marker is still challenging. Indeed, there is no sound conclusion on the biomarkers as the inconsistent data reported about the changes in DNA methylation in different genes, different sample types and different regions (Nikolac Perkovic et al., 2021). Given that the literature related to OPRD1 DNA methylation in AD is limited, continuous research on large number of AD specimens with different types or brain samples in different regions is required. Until now, no information concerning specific OPRD1 mutation has been observed in AD pathologies neither before nor after the onset of clinical symptoms. These concerns also restrict the development of OPRD1 or DOR as a biomarker for AD diagnosis.

Building upon our preliminary finding of a significant negative correlation between DOR and C1q in the brain tissue of AD mouse model and microglia (Xu et al., 2025a), converging evidence underscores the translational potential of this axis as a biomarker. Complement C1q has emerged as a promising diagnostic marker across dementia subtypes, supported by studies showing elevated CSF C1q correlating with complement activation in genetic frontotemporal dementia, even potentially prior to symptom onset (van der Ende et al., 2022; Veteleanu et al., 2023). Bioinformatic and experimental analyses identify C1q subunits (C1qa, C1qb, C1qc) as hub genes markedly upregulated in vascular dementia models and patient serum, with C1qb demonstrating significant diagnostic capacity (*AUC* = 0.799) (Xu et al., 2025c). Moreover, plasma exosomal C1qc is part of a protein signature that accurately discriminates AD patients from controls and correlates with cognitive decline (Cai et al., 2022). Given this compelling evidence for C1q's role and its stringent inverse relationship with DOR, we propose that a combined strategy to detect both the molecules could substantially enhance diagnostic precision. Since recent studies have confirmed the expression of functional DOR in peripheral blood mononuclear cells (PBMCs), with detectable mRNA and protein levels reported in circulating lymphocytes and monocytes (Brejchova et al., 2020; Eisenstein, 2019), the membrane-bound nature of DOR may offer a practical and minimally invasive platform for dual-analyte assessment. However, we acknowledge several limitations of this combined strategy. First, DOR expression in PBMCs is generally lower than in neural tissues, necessitating the adoption of highly sensitive quantitative approaches, such as high sensitivity chemiluminescence, to ensure reliable and reproducible detection. Second, this detection strategy requires careful optimization of sample collection, storage, and processing, as receptor degradation during freeze-thaw cycles may lead to inaccurate quantification. Third, several confounding factors, including acute infections or metabolic disorders, could independently alter PBMC receptor profiles (Reyes et al., 2020; Hu et al., 2024). Therefore, rigorous inclusion/exclusion criteria and standardized operating procedures are critical to isolate the AD-specific DOR-C1q signature. On the other hand, recent advances in multiplex immunoassays and flow cytometry have enabled simultaneous quantification of low-abundance membrane receptors alongside complement factors in minimal blood samples (Carvelli et al., 2020; Leipold and Maecker, 2012), supporting the technical feasibility of our proposed dual-biomarker approach. This combinatorial approach is hypothesized to improve specificity and sensitivity over single-marker assays by capturing a more comprehensive pathophysiological signal which reflects both neuroinflammatory complement activity (via C1q) and its putative regulatory counterpart (via DOR). Beyond diagnosis, longitudinal monitoring of the DOR-C1q ratio in PBMCs could serve as a dynamic pharmacodynamic biomarker, offering a valuable tool for stratifying patients, evaluating therapeutic efficacy, and tracking disease progression in clinical trials for AD and related dementias.

## 7 DOR as a target for the treatment of neuropsychiatric symptoms of AD

Beyond the treatment of AD pathologies and cognitive impairment, progress is being made to develop intervention drugs for the neuropsychiatric symptoms in AD and dementia. Agitation is a common problem in AD, occurring in majority of patients during the disease course (Scheltens et al., 2021; Koenig et al., 2016). Together with agitation, depression was also implicated in AD pathogenesis. A prolonged depression in late life could be a precursor or symptom to dementia in AD (Huang et al., 2024). Several selective DOR agonists have been proved to have great potential in anxiolytic and antidepressant (Yoshioka et al., 2023; Dripps et al., 2020; Olson et al., 2023; Jelen et al., 2022; Moriya et al., 2023; Chen et al., 2022; Wu et al., 2023). Early in 2000, (Filliol et al., 2000) investigated OPRD1-deficient mice exhibited constantly anxiogenic and depressive-like responses, highlighting the beneficial role of DOR in emotional regulations. The research group from University of Michigan identified diprenorphine as a safe and fast-acting antidepressant drug (Olson et al., 2023). Diprenorphine is a partial agonist of DOR and κ-opioid receptor, and an antagonist for MOR. Studies in C57BL/6 mice have well demonstrated that an acute dose of diprenorphine could induce antidepressant-like effects in the tail suspension test (TST) and the novelty-induced hyperphagia test, while these effects were abolished by DOR antagonist naltrindole (Olson et al., 2023). Indeed, some studies have demonstrated that DOR mediated antidepressant-like effects are independent from MOR induced mood regulations. In that work, MOR knockout (KO) mice and wildtype (WT) mice were treated with KNT-127, a selective DOR agonist, respectively. The mood status was evaluated by the immobility time of mice during the TST and forced swim test (FST) test (Moriya et al., 2023). As a result, both MOR knockout and KNT-127 administration significantly decreased immobility time of mice in both tests. Treating MOR-KO mice with KNT-127 further decreased the immobility time of female MOR-KO mice in the TST test, while it was not effective in male MOR-KO mice. The antidepressant-like effects of DOR agonist SNC80 were also validated in various of animal models (Chen et al., 2022). Chen et al. found that SNC80 dose-dependently produced antidepressant-like effects in rats under normal state, and this effect was enhanced when rats suffered from inflammatory pain (Chen et al., 2022). Wu et al. examined the anti-depressant effects of SNC80 in mice subjected to chronic-restraint-stress (CRS) by TST, FST and sucrose preference test (SPT). The findings showed that SNC80 significantly reversed depressive-like behaviors by upregulating the BDNF/TrκB signaling pathway (Wu et al., 2023). Deltorphin (DLT) and deltorphin II (DLT-II) have high affinity and selectivity for DOR. Intracerebroventricular administration of DLT in mice exerted antidepressant-like effects during TST test, which could be counteracted by DOR antagonist 7-benzylidenenaltrexone (Nakagawasai et al., 2022). More recently, KK-103, a prodrug of leucine-enkephalin (Leu-ENK), was developed (Hohenwarter et al., 2024). Compared to the high proteolytic instability properties of Leu-ENK as an endogenous DOR agonist, KK-103 is more stable with the similar ability to antidepression, but with minimal gastrointestinal inhibition and no incidence of sedation (Hohenwarter et al., 2024).

Despite these promising preclinical findings, the translational potential of DOR agonists for AD patients must be weighed against several critical safety and pharmacological concerns. First, central nervous system (CNS) penetration is a prerequisite, and optimal candidates must cross the blood-brain barrier effectively to achieve central efficacy while minimizing peripheral adverse events. Second, long-term safety and tolerance in the aging brain require rigorous investigation. Although DOR agonists lack the typical MOR abuse liability, chronic exposure may still trigger receptor desensitization or internalization, leading to progressive loss of efficacy. Third, potential adverse effects cannot be overlooked. Some selective agonists such as SNC80 have been reported to induce convulsions at higher doses, which poses a significant challenge for AD patients with upregulated seizure susceptibility (Xia, 2015). While newer agents like KK-103 show improved BBB penetration and reduced gastrointestinal and sedative effects, a comprehensive safety profile regarding cardiovascular risks, sedation, and gastrointestinal motility in geriatric populations requires thorough evaluation (Hohenwarter et al., 2024; Viswanadham et al., 2021). Thus, future drug development should prioritize pharmacokinetic optimization and rigorous toxicological screening to establish a favorable risk-benefit balance for this vulnerable patient population.

A growing body of evidence indicates the direct involvement of DOR in the regulation of mood and its potent capacity in antidepression without abuse liability (Adzic et al., 2023). This has led researchers to investigate their agonists or ligand as adjuvants or alternatives to traditional antidepressants. Indeed, the recent findings have indicated DOR modulators as a more effective therapeutic strategy than conventional medications for depression treatment in elderly with combined mild cognitive and depressive-anxiety disorders (Adzic et al., 2023).

## 8 Conclusions and perspectives

In the past decades, substantial efforts have been made to promote the diagnosis and treatment of AD. DOR has been identified by emerging evidence from various animal models as a promising target for Aβ clearance, tau dephosphorylation, microglial modulations and neuropsychiatric symptoms treatment, as well as diagnosis of AD (Table 2). However, the mechanistic studies of DOR's action on AD pathology are still insufficient. Also, the genetic studies are incomplete with a lack of pinpoint whether DOR mutation occurs in AD process as a risk gene.

**Table 2:** Summary of main findings from *in vitro* and *in vivo* studies investigating DOR modulation in Alzheimer's disease and related models.

References	Model system	DOR agonist/antagonist	Key findings	Pathological domain
*In vitro* studies				
Ni et al. (2006)	C99/DOR-transfected HEK293 cells; primary hippocampal cultures	DADLE (agonist)Naltrindole (antagonist)	DOR activation increased Aβ production and secretion, likely by facilitating γ-secretase-mediated cleavage of the Aβ precursor C99	Aβ regulation
Teng et al. (2010)	C99/DOR-transfected HEK293T cells	DADLE (agonist) Naltrindole (antagonist)	DOR activation promoted the formation of a DOR-BACE1-γ-secretase complex and enhanced APP processing through trafficking into late endosomes/lysosomes	Aβ regulation
Leskelä et al. (2009, 2012)	SH-SY5Y; HEK293 cells	DOR Cys27 loss-of-function variant (genetic)	The DOR Cys27 variant impaired receptor maturation and surface trafficking, and increased APP processing and Aβ generation	Aβ regulation
Xu et al. (2020)	Aβ oligomer-treated PC12 cells; APPswe SH-SY5Y	UFP-512 (agonist)Naltrindole (antagonist) DOR knockdown	Selective DOR activation reduced BACE1 expression/activity, attenuated APP cleavage, and decreased Aβ generation. These protective effects were reversed by DOR antagonism, whereas DOR knockdown increased BACE1 activity and accelerated APP processing	Aβ regulation
Li et al. (2025)	Okadaic acid-induced tauopathy model in highly differentiated PC12 cells	UFP-512 (agonist)Naltrindole (antagonist)	DOR activation dose-dependently reduced tau hyperphosphorylation at Thr231 and Ser262, downregulated CDK5 and AMPK phosphorylation, and reduced CyclinD1, CyclinB1, and cleaved caspase-3	Tau pathology/neuronal injury
Cheng et al. (2021)	BV2 microglia	Tan67 (agonist)Naltrindole (antagonist)	DOR activation suppressed LPS-induced inflammatory responses in BV2 microglia, including reduced pro-inflammatory cytokine release, partly through inhibition of the MAPK/caspase-3 pathway	Neuroinflammation
Xu et al. (2022)	BV2 microglia	UFP-512 (agonist)Naltrindole (antagonist)	DOR expression was upregulated by IL-4 and hypoxia but downregulated by LPS and Aβ oligomers. UFP-512 suppressed BV2 activation and inflammatory responses under LPS, hypoxic, and AD-like injury conditions, whereas DOR antagonism enhanced microglial activation	Neuroinflammation
Xu et al. (2025b)	BV2 microglia	UFP-512 (agonist);DOR overexpression/knockdown	DOR overexpression enriched microglial homeostatic genes and downregulated disease-associated microglia genes, whereas DOR knockdown produced the opposite effect. DOR activation also inhibited HMGB1/NF-κB signaling.	Neuroinflammation/microglial activation
Xu et al. (2025a)	BV2 microglia; microglia-neuron coculture	UFP-512 (agonist);DOR overexpression	DOR was identified as a novel classical complement pathway inhibitor through interaction with microglia-derived C1q. DOR activation or overexpression promoted DOR-C1q complex formation, restrained classical complement pathway activation, reduced microglial phagocytosis, and rescued synaptic proteins	Synaptic pruning/neuroinflammation
Min et al. (2018)	Cerebral ischemia/reperfusion mouse model	Tan-67 (agonist)Naltrindole (antagonist)	Post-ischemic DOR activation initially increased APP expression, maturation, and processing within 6 h after ischemia/reperfusion, but later reduced BACE1 expression/activity by 24 h and decreased Aβ production. These effects were abolished by naltrindole.	Aβ regulation
Xu et al. (2025b)	APP/PS1 mice (9-month-old)	UFP-512 (agonist)	UFP-512 treatment sharply reduced insoluble Aβ42 levels in cortex and hippocampus and decreased Aβ plaque burden. It also reduced neuroinflammation, microglial activation, and macrophage infiltration in the AD mouse brain.	Aβ regulation/neuroinflammation
Sawada and Yamakage (2024)	Pregnant C57BL/6JJmsSlc female mice	Endogenous DOR upregulation associated with pregnancy-induced analgesia	Pregnancy-induced analgesia was associated with increased DOR expression in the anterior cingulate cortex and suppressed microglial activation in late-pregnant mice.	Neuroinflammation/microglial activation
Filliol et al. (2000)	OPRD1-deficient mice	Genetic knockout of DOR	DOR deficiency induced anxiogenic and depressive-like responses	Neuropsychiatric symptoms
Olson et al. (2023)	C57BL/6 mice	Diprenorphine (DOR partial agonist)Naltrindole (antagonist)	Diprenorphine produced antidepressant-like effects in the tail suspension test, and these effects were abolished by naltrindole	Neuropsychiatric symptoms
Moriya et al. (2023)	MOR-knockout and wild-type mice	KNT-127 (agonist)	KNT-127 reduced immobility time in tail suspension and forced swim tests, supporting antidepressant-like effects mediated by DOR activation	Neuropsychiatric symptoms
Wu et al. (2023)	Chronic-restraint-stress mice	SNC80 (agonist)	SNC80 reversed depressive-like behaviors and increased BDNF/TrkB signaling.	Neuropsychiatric symptoms
Nakagawasai et al. (2022)	Male ddY mice	Deltorphin/deltorphin II (agonist)7-benzylidenenaltrexone (antagonist)	Deltorphin and deltorphin II induced antidepressant-like effects in the tail suspension test, which were counteracted by DOR antagonism	Neuropsychiatric symptoms
Xu et al. (2025a)	APP/PS1 mouse model	UFP-512 (agonist) Naltrindole (antagonist)	DOR activation reduced C1q levels, restrained classical complement pathway activation, attenuated microglia-mediated synaptic pruning, and rescued synaptic loss in AD pathology	Synaptic pruning

Moreover, translating these promising preclinical findings into clinical practice requires critical consideration of several translational barriers. The first is polypharmacy, which is common among AD patients and raises concerns regarding potential pharmacodynamic interactions. For example, as several psychotropic drugs and DOR agonist are CNS-active, their concurrent use may increase the risk of cognitive impairment and fall-related injuries (Maust et al., 2021). Hence, a comprehensive assessment of these drug-drug interactions is particularly important before clinical application.

Second, the dual and context-dependent nature of microglial activation should be rigorously considered. Although DOR activation may attenuate AD-related neuroinflammation by inhibiting HMGB1-NF-kappaB signaling and the MAPK-caspase-3 axis, thereby suppressing pathological microglial activation, DOR-mediated signaling is unlikely to be uniformly protective across disease contexts. In particular, DOR activation has also been reported to exert antidepressant-like effects through upregulation of the BDNF-TrkB pathway in depression models (Wu et al., 2023). This raises an important safety concern, because microglial activation can also be triggered by BDNF-TrkB signaling and may contribute to nerve injury under certain pathological conditions (Hu et al., 2023). Furthermore, vascular deterioration is commonly observed in patients with AD and increases the risk of intracerebral hemorrhage and ischemic events (Santisteban et al., 2022). Recent evidence suggests that ischemia can promote microglial activation through the BDNF-TrkB cascade, accompanied by alterations in GABAergic and glutamatergic neurotransmission (Zhou et al., 2026). Therefore, in AD patients with cerebrovascular vulnerability, DOR agonist-induced or DOR-associated enhancement of BDNF-TrkB signaling could have unpredictable consequences, despite its potential benefits in other pathological and neuropsychiatric aspects.

Third, the acute and long-term adverse effects of DOR agonists warrant thorough preclinical safety profiling. For example, simultaneous inhibition of HMGB1-NF-κB and MAPK-caspase-3 signaling, together with activation of BDNF-TrkB signaling in microglia, may produce complex and potentially adverse effects on microglial phenotype, neuronal survival, and synaptic homeostasis. In addition, poor ligand specificity for DOR may lead to off-target interactions with MOR, KOR and other opioid receptor subtypes, increasing the risk of unwanted opioid-related side effects. Therefore, future drug development should prioritize target-selectivity optimization, rigorous toxicological evaluation, cerebrovascular safety assessment, and multidrug interaction studies to ensure a favorable risk-benefit profile for this vulnerable patient population.

The continued work to understand the role of DOR in AD pathogenesis and genetic basis will probably lead to a complete rethinking of the relationship between DOR and AD and provide a whole new idea on AD diagnosis and therapy.
